# Reviews and Book Notices

**Published:** 1879-05

**Authors:** 


### Reviews and Book Notices.

---

ARTICLE XVI.

A PRACTICAL TREATISE ON DISEASES OF THE SKIN. By Louis A. Duhring, M.D., Professor of Diseases of the Skin in the Hospital of the University of Pennsylvania, etc., etc. Philadelphia: J. B. Lippincott & Co., 1877.

AN ATLAS OF SKIN DISEASES, By Louis A. Duhring, M.D., Professor of Skin Diseases in the Hospital of the University of Pennsylvania, etc. Part I, Eczema (Erythematosum), Psoriasis, Lupus Erythematosus, Syphiloderma (Pustulosum). Part II, Acne Rosacea, Ichthyosis (Simplex), Tinea Versicolor, Sycosis (Non-Parasitica). Part III, Eczema (Squamosum), Syphiloderma (Erythematosum), Purpura (Simplex), Syphiloderma, (Papulosum et Pustulosum). Part IV, Vitiligo, Alopecia Areata, Tinea Favosa, Eczema (Rubrum).

A HANDBOOK OF THE DIAGNOSIS OF SKIN DISEASES. By Robert Liveing, A.M. and M.D., Cantab., F.R.C.P., London, etc., etc.: New York, William Wood & Co., publishers, 1879.

NOTES ON THE TREATMENT OF SKIN DISEASES. By the Same. Fourth Edition, revised and enlarged. New York: William Wood & Co., publishers, 1879.

It is usually the part of the critic and reviewer to pronounce, in advance of the public, upon the merits or demerits of the work submitted to him. It is true that his decision, one way or the other, may have but little to do with the success of an author, as compared with that eagerness for everything possessing real value, which is, sooner or later, sure to manifest itself on the part of the reading public. Still the critic has usually the immense advantage of the master of ceremonies at the formal introduction.

In the present instance, this usual order is reversed. The book before us has been long since presented to the public, and passed upon without reserve by the members of the medical profession. Fortuitous circumstances, which it is not here necessary to describe, have thus far prevented us from doing justice, in the pages of this journal, to the book itself, or to its considerately patient author and publishers. At this late date, it merely remains for us to record that seal which has been already set upon Duhring's treatise, by worthier and more competent judges.

Needless to say, here, that that seal has been one of approval. We might well, at this point, conclude all that remains to be said upon the subject, by quoting the recorded verdict of the press, that this is "the best treatise on diseases of the skin in the English language." But lest this should be thought the exaggeration of personal friendship or favoritism, we are ready to give the grounds for our opinion.

Every work of art of high order, is so intrinsically good that its excellences have to be ascertained by analysis. The volume before us is fully as valuable for what it is not, as for what it is.

We remark, then, that it is an original treatise and neither a compilation of previously published sketches nor a series of clinical observations, nor an argument upon one side of a disputed question, nor yet an ingenious mosaic of the opinions and writings of other men. It is a systematic treatise, without either the diffuseness or incongruity of so many of its predecessors. It is a carefully written book, without that absurdity of style, half conversational—half declamatory, which is so offensively prominent in an English author upon the same subject, whose book has attained some prominence in this country. Lastly, it is preeminently a book for the student and the practitioner. From it they can acquire the elementary as well as the practical and the scientific knowledge, that are essential to the successful recognition and treatment of diseases of the skin.

Dedicated to the eminent Hebra, of Vienna, we are prepared to find that the work of our author largely represents the views of the German school, both as to the anatomico-pathological classification of the diseases of the skin, and as to the mode of their treatment. In both respects, however, the modifications here effected are on the side of a conservative recognition of those diseases which are unquestionably of constitutional origin, while the extreme opinions of the French and a few English and American authors, who hold to the existence of "dartres" and "rheumides," and who look upon eczema and psoriasis as evidence of a diathetic vice, are unreservedly rejected.

The clearness and simplicity resulting from the embodiment in these pages of only those facts which are capable of clinical demonstration, give them a charm and value which constitute the basis for the actual success of the book. Take, for example, the subject of impetigo:—

Willan and Bateman were early in the dust and confusion about this term. What time they confounded impetigo with porrigo, and the reverse, they described six varieties of each disease. Alibert, as is well known, in his early classification described the disease, first, in connection with acne, as a "pustular dartre," afterward as a "pustular crusted psoride"; and in his later classification it reappears under successive titles, as an "eczematosis," a "phlyzacion," a "melitagra," and a "tinea." Cazenave and Schedel upon this point say:

"The eruption (impetigo) is pustular from the beginning, and the pustules are small, confluent, and very slightly raised above the level of the skin. They burst between thirty-six and seventy-two hours from their formation, and discharge a purulent fluid. The heat, itching and tension are then increased. The fluid is abundantly discharged by numerous small orifices; it soon dries up, and forms thickish, yellow, friable, semi-transparent incrustations, which have some resemblance to the gummy exudations of certain trees, or to layers of concrete honey."

There are few men, even with the most superficial knowledge of dermatology, who will fail to recognize in this description the features characteristic of a well-marked form of eczema. And yet they do not "do these things better in France" to-day. Thirty years after this paragraph was penned, Guibout writes in Paris as follows (1876):

"Impetigo is characterized by the existence of small, shortlived fugacious, acuminate, confluent pustules, seated upon a more or less extensive erythematous surface, secreting a pus which continues to be formed after their disappearance, by ulcerated surfaces which succeed them, and which conclude by forming moist, yellow, projecting, irregular crusts, of the color of honey or marmalade of apricots."

Hebra, as is known, entirely rejects the term, impetigo, from his classification of diseases of the skin. In a chapter entitled "pustular eruptions," he gives fully his reasons for this course, and, if he had never done anything else for dermatology, he would have deserved the honor he has been accorded, for his masterly treatment of this subject alone. He recognizes the fact that the pustule is a resultant of an inflammatory process; that the inflammatory process we call, eczema, may result in the formation of a pustule, or a puriform discharge; and that the existence of a pustule upon the skin, is no evidence whatever, *per se*, of the existence of any special disease, since it may be the indirect result of the bite of a louse, or of the external application of croton oil. In his own language: "the pustular affections of the skin described by authors, under the names, impetigo, ecthyma, porrigo, achor, etc., have no existence as independent diseases."

We have made this short excursion merely for the purpose of showing how simply, and yet how admirably, one author leads us straight through this forest of perplexities, by a sure path. Under the head of "pustular exudations," we find the word, impetigo, in Duhring's classification; and the disease in proper place, is thus defined: "Impetigo is an acute, exudative disease, characterized by one or more pea or finger-nail sized, discrete, rounded and elevated, firm pustules, unattended by itching, occurring, for the most part, in children." The four pages of descriptive text which follow, portray in detail a disease whose identity is demonstrable by daily clinical experience, and which is assuredly very different from eczema rubrum or eczema pustulosum. The lesions are pustular at the outset, have a slight areola, are not umbilicated, are constituted of the entire thickness of the epidermis, *have no disposition to rupture*, are discrete, vary in number from a dozen to more, are not attended with either itching or burning, as a rule, and their contents may undergo reabsorption or crusting. Surely the most casual reader of these lines, can here follow the keen and practised eye of a master, who has the courage to depart from under the shadow of the high authority of the great Vienna dermatologist without wandering into the mazes and mysticisms of the French school.

In order to make our picture more complete, let us turn for a moment to Duhring's description of eczema pustulosum, where we shall find a history of the exact lesions, whose shadowy ghosts have been flitting for years in the pages of Willan and Bateman, Cazenave and Schedel, Biett, Alibert, Guibout, and many others. The text is as follows:

" The pustules are formed in the same manner as the vesicles which have been described, except that there is not the same amount of swelling, heat and itching present. The pustules are apt to be somewhat larger than the vesicles, and are, as a rule, firmer in consistence. They develop as pustules, or, as is often observed, they become pustules from vesicles; again, both lesions may exist at the same time, side by side, upon one individual. A strict line cannot be drawn between vesicles and pustules. As in the case of the vesicles, the pustules burst, and are replaced by thick, bulky, greenish, yellow or brownish crusts, which are apt to cover the skin completely. If this process continue, they accumulate in quantity, causing great disfigurement. They desiccate quickly, and become friable, and finally fall off or crumble away."

And thus might we discourse of each of the topics in the treatise. In connection with each is to be noted that same exactness of definition which results from distinctness of conception, the same avoidance of ambiguity and unsettled or disputed questions, the same straightforward progress over a well-built roadway, upon which the traveler passes without dreaming of the wrecked theories which are lying at the bottom.

The style of the author is conspicuously clear and pleasant, for the same scholarly care which has been made evident in his definitions, is here to be recognized. The same is true of the typographical appearance of the work, which, as is well understood, is in this respect quite singular. During the months which have elapsed since they were first given to the profession, we have frequently consulted these pages, and have never yet detected an error in typography or syntax. The plates are well drawn, and the publishers have well executed their part in the production of the treatise.

We conclude, as we began, by expressing our conviction that this is the best treatise on diseases of the skin in the English language, judged from every standpoint. We are even willing to say more than this. Well would it be for the medical literature of our land, if the standard of even its best representatives might never be less than that evidently upheld by our young and gifted author.

DR. DUHRING'S ATLAS OF SKIN DISEASES is a fit companion of the other work of his hand. We have, upon another occasion, referred in these pages to the excellence of these plates, and can here no more than iterate our former praises.

The faithfulness with which the disease is, in each instance, portrayed, is due to no fortuitous circumstances. Like every other worthy accomplishment, it is the fruit of the author's careful and conscientious labor. From the selection of the individual case whose lesions are to be reproduced, to the work on the canvas (where the author frequently applies the finishing touches with his own hand), to the multiplication of the stones (the lithographer having a *carte blanche* order to employ as many as are necessary in order to reproduce exactly the different colors of the original picture), and even to the actual printing of the final impressions, each and every step is under the same watchful supervision and superintendence. It is in consequence of this care and expenditure of time and money, that the plates are equal to any that have yet been published.

The explanatory text accompanying each, is a clear and sufficiently brief exposition of the case selected for illustration, and the treatment pursued, with its results.

The only adverse criticism we have yet seen of this work may be adduced in support of its extraordinary value. An English writer, in reviewing Dr. Duhring's atlas, complained that, while the author had ostensibly undertaken to represent distinctively

American types of cutaneous disease, his plates yet represented quite exactly the individuals effected with eczema, acne, etc., who were to be encountered every day among the patients and out-patients of the London hospitals. Surely no higher commendation than this could be pronounced. While it may be true that the prurigo, xeroderma, pityriasis rubra, lichen scrofolosus, and some other of the diseases seen in the Vienna wards, are rare in America, it is equally probable that the disorders of the skin observed in the two great English-speaking countries, are more similar than those of any other two widely separated people.

As the stones from which these admirable plates are printed, are to be eventually destroyed, it is evident that the value of this atlas will increase with each year.

DR. LIVEING'S SMALL VOLUMES are sufficiently compact to be stowed into the pocket of the student as he goes through the wards of a hospital, or attends a dermatological clinic. The aim of the author has been, "to call attention to the peculiarities and distinctive points which enable us to form a sound and rapid diagnosis." It is a question with us, however, whether a "rapid diagnosis," can be generally considered "sound," in actual practice. And again, has not he who is capable of making a sound and rapid diagnosis, usually advanced far beyond the teachings of the manuals of this class; while the student confronted with his first case of skin disease, is usually incapable of making any diagnosis whatever?

Books of this sort remind us ever of the doll-houses seen in the windows of the toy-shops. As one inspects them, he finds they are thoroughly furnished in all particulars. Each article too, is in its right place. Everything requisite for the *ménage* is there in miniature. The only objection to the whole, is that it is *too small to be practically useful.*

We remark, however, that Dr. Liveing's books exhibit a commendable familiarity with the subjects under discussion, a familiarity with the writings of even American authors, which has until lately, been rarely noted among our English cousins. The little volumes will prove popular with that class of practitioners, who desire to find their literature of this subject compressed in the smallest possible compass. J. N. H.

ARTICLE XVII.—MEDICAL AND SURGICAL ELECTRICITY. By Beard and Rockwell. William Wood, & Co., 1878.

There is probably no American work on electricity that has received the approval of the profession to such an extent as has this work. In the first edition of this work there were faults which were open to severe criticism, but most of which have been corrected in this new edition. New chapters have been added, and the whole work has been so thoroughly revised, that the physician who employs electricity in the treatment of disease can ill afford to be without it. The work is divided into the following sections: Electro-physics, Electro-physiology, Electro-therapeutics and Electro-surgery.

Under the first division the physics of electricity are given as exhaustively as any medical man would require. On page 2, this statement is made: " Electricity is manifested in three general forms: *magnetism*, *statical* or *frictional* or *Franklinic* electricity, and *galvanism* or *voltaic* or *dynamical* electricity." On page 34 it is stated that: " If we start with magnetism, we find that it produces electricity, and through electricity, heat, chemical action and light." * * * " If we start with electricity, we find that it produces magnetism, heat, light, chemical action and motion." In the first statement, electricity and magnetism are held to be one and the same force, or rather that magnetism is a form or manifestation of electricity. In the second, it is reasonable to infer from the text, that they are held to be different forces, just as light and heat are different. Although electricity and magnetism are more nearly co-related than the other forces, and have much in common, we are not inclined to accept these forces as being one and the same on the evidence already adduced; notwithstanding some eminent physicists think the time is not far distant when they will be proven to be one.

The statement: " The popular notion that large cells have a therapeutic advantage over small cells by sending a larger quantity of electricity through the body, is, in the light of Ohm's law, as well as in the light of experience, erroneous," is well worth the attention of physicians.

Beard and Rockwell do not stand alone in this statement.

Poore—one of the more recent English authors—makes a similar assertion.

They also state that: " It follows that the dose of an electrical application cannot be accurately described by stating the number of cells and the length of the sitting." " Supposing, now, that we are treating a patient locally or centrally by the galvanic current, and we desire to transfer the patient to another physician; we inform the physician to whom the transfer is made, that we are treating the patient with ten cells for ten minutes, and we desire that he should continue to give the same dose. * * * In our very attempt to be accurate we stumble into gross inaccuracy. Had we left the whole matter to the judgment of the physician, with some general suggestions as to the susceptibility of the patient, we should have come far nearer the truth." The fallacy this assertion points out and attempts to correct, is a fallacy that physicians little acquainted with the application of electricty are apt to take up, and is one that all thinking electro-therapeutists have many times demonstrated, to their own satisfaction, to be as untrue as our authors make it. Beard and Rockwell insist with equal truth that the efficacy of an electrical treatment depends as much or more on the mode of administration as on the dose of electricity administered.

The authors lay great stress on the necessity of a complete and thorough knowledge of the construction of the apparatus as well as the chemistry of the battery—a fact well worthy the attention of all who employ electricity.

Electro-physiology is also very fully treated of, and cannot fail to be of interest and benefit.

Under the head of electro-therapeutics, the history of electrical applications, electro-diagnosis, the apparatus for therapeutic use, and the treatment of disease, are given.

This important division occupies more than one-half of the volume. Various cases from practice are given to illustrate the modes of treatment in individual cases. A full and exhaustive review of all of the scientific methods of electrical treatment is also given.

Among the many excellent suggestions found in the chapter on " General suggestions," we cull the following: " Electricity is a powerful *stimulating sedative tonic.*" "The old notion that electricity was merely a stimulant aided in forming in the professional mind another very gross error, that in active inflammations electricity is contra-indicated. Experience proves every day that the sedative effects of electricity are exceedingly grateful even in the acute stages of sprains and diseased joints." "The best results of electrical treatment are usually obtained with mild currents," and "Even at this advanced stage of electro-therapeutics, it seems to be necessary to constantly warn the profession against indiscriminately intrusting the details of electrical applications to the nurses, friends of patients, and patients themselves. Having just rescued this department from the hands of the laity, and given it a position among men of science, it seems strange that those physicians who are familiar with the subject, should even now use their influence to return it to the people at whose hands it formerly suffered so much; to restore it to the captivity of prejudice and ignorance." The limits of this review warn us that we have măde as full extracts as would be proper, and those who wish farther light on the subject must read the book for themselves.

In this edition is introduced a chapter on "central galvanization"—a most valuable method of employing galvanic electricity in certain forms of nerve-exhaustion, skin diseases, etc.

The section on Electro-surgery treats of electrolysis and the galvano-cautery.

In reading this book, we have found many facts stated, which every physician, who has had a large experience with electricity, has discovered, and which he may think, as we did, were unknown until we read the statement of facts in these pages. There is probably no work in the English language, which deals with this subject, that covers so much ground and is so free from error as is this work. It is thus fitted in every respect to be a standard work on medical electricity.

Before leaving the subject, we can but in justice refer to some of the new methods of electrical treatment and of electro-physics as related to physiology and therapeutics, which these authors were the first to present to the medical profession. They have explained Ohm's law in reference to physiology and therapeutics, have originated "general faradization," "central galvanization," have shown the efficacy of the galvanic current in the treatment of skin diseases, and in electro-surgery of that method known as "electrolysis of the base."

P. S. H.

ARTICLE XVIII. — ATLAS OF DISEASES OF THE MEMBRANA TYMPANI. By H. Macnaughton Jones, M.D., etc. Philadelphia: Lindsay & Blakiston, 1878; Chicago: Jansen, McClurg & Co.

This atlas, the author tells us, is intended as an accompaniment to his treatise on Aural Surgery, and the description of the diseases illustrated is, with some slight modifications, taken from that work. The atlas contains six chromo-lithographic plates of nine figures each, intended to illustrate the membrana tympani and some of its diseases and two plates, one of four and the other of five drawings of diseases of the external ear.

Were we to judge of the merits of this work from the author's own estimate of it, as expressed in his really gushing preface, we certainly should have little to criticise. The great diversity of appearance that the membrana tympani may present in each of the affections involving this structure, renders it difficult to say whether the drawings of the diseased conditions here presented, are faithful representations or not. But the author has given us a drawing of a normal membrane (fig. 6), and this should be a standard by which to estimate the value of the other drawings. Let us examine it. According to the author's own description, "the healthy membrana tympani varies in color from a 'pearl' or 'neutral gray,' to a yellowish white, or at times even an ivory white." His drawing of the normal membrane is neither of these shades, but a light blue. If the drawing is intended for the left membrane, the long diameter is shown as being in the direction backwards and downwards. The handle of the malleus appears as occupying the middle third of the long diameter. What we suppose is intended to represent the short process of the malleus, is about as far from the anterior and upper edge of the membrane as the tip of the malleus is from the posterior and lower edge. No trace of the anterior ligament of the malleus is shown. We are glad the author has told us that figure 6 represents a normal membrane, for we certainly would not have recognized it as such.

Fig. 31, " Old Eustachian case. Membrane very white, etc." If the membrane in this figure is white, then we must be color-blind, for it does not appear so to us.

Fig. 55, an abscess in external meatus. Fig. 56, Polypus protruding from meatus. It would be difficult to tell from the drawings which is the abscess and which the polypus.

The chromo-lithographs may do credit to artists in that line in Cork, where according to the author, there was the first attempt to lithograph drawings. In this country where the chromo is offered as a premium or inducement to purchase anything, from a pound of tea to a corner lot, the art has made greater progress, and our standard of creditable work is much higher. An atlas of aural diseases, is to the student of these diseases, what the map of a country is to the student of the geography of that country. It aids him if correct, but confuses and misleads him if incorrect. In our opinion the work before us comes under the latter clause. W. T. M.

ARTICLE XIX.—DISEASES OF THE BLADDER AND URETHRA IN WOMEN. By Alexander J. C. Skeene, M.D., Professor of the Diseases of Women in the Long Island College Hospital; Fellow of the American Gynæcological Society; Corresponding Member of the Gynæcological Society of Boston; Member of the Medical Society of the County of Kings, and of the Obstetrical Society of New York. New York: Wm. Wood & Co., 1878; pp. 375.

The author divides this book into eight chapters, their subjects being as follows:

*Chapter I.*—Anatomy of the Bladder and Urethra, and their Malformations.

*Chapter II.*—Functional Diseases of the Bladder. Irritability. Paresis. Ischuria. Enuresis. Disorders due to Diseases of other Pelvic Organs. Disorders from Anomalies of Form and Position of Bladder. Extroversion through the Urethra.

*Chapter III.*—Organic Diseases of Bladder. Urinary Analysis and Exploration of Bladder. Hyperæmia. Hæmorrhage.

*Chapter IV.*—Cystitis, Sub-acute, Chronic, Catarrhal, Interstitial, Croupous, Diphtheritic, Gonorrhœal, and Peri and Epi-Cystitis. Etiology, Pathology and Symptomatology.

*Chapter V.*—Treatment of Cystitis. Croupous and Diphtheritic Cystitis. Cystitis with Epidermoid Concrement. Vesico-Urethral Fissure.

*Chapter VI.*—Neoplasms, Cysts, Tubercle and Carcinoma of the Bladder. Foreign Bodies. Hypertrophy and Atrophy and their Etiology, Pathology, Symptomatology and Treatment.

*Chapter VII.*—Urethral Diseases. Neuroses. Urethritis, Acute, Chronic and Gonorrhœal, Circumscribed and Sub-acute. Neoplasms. Vascular Tumors. Areolar, Epithelial and Compound Neoplasms and their Symptoms, Diagnosis, Etiology, Prognosis and Treatment.

*Chapter VIII.*—Urethral Dilatations and Dislocations. Prolapsus of the Mucous Membrane. Foreign Bodies in Urethra. Stricture. Incomplete Fistula in Urethra.

We can heartily commend this work to physicians. A medical library is not complete without it, because it seems to be the best work on the subjects treated, now before the profession.

J. H. E.

ARTICLE XX. — ON THE SURGERY OF THE FACE. By Francis Mason, F.R.C.S., Surgeon and Lecturer on Anatomy at St. Thomas' Hospital, etc. With one hundred illustrations. Philadelphia: Lindsay, Blakiston & Co., 1879. Chicago: Jansen, McClurg & Co., pp. 170; price $2.25.

This volume comprises the three Lettsomian lectures delivered by the author in the early part of last year, and at the time published in the *London Lancet.*

The first lecture is devoted to the diseases, the second to injuries, and the third to the deformities of the face.

We cannot expect that this subject could be exhausted in three lectures, nor had the lecturer any such object in view. He rather intended to give a brief review of the whole field; but his vast experience lends to every page the attractions of a mature judgment. We will content ourselves with a single quotation in order to give our readers an idea of the author's style of writing.

Speaking of the operation of hare-lip, he condenses volumes of instruction on plastic surgery in the following remarks (p. 102): "The hare-lip pin with the twisted suture, is commonly employed to bring the edges together, but, without discarding this method of approximating the parts, I feel sure that the usual interrupted suture of silk may in most cases be employed with great advantage, and, I believe, with certain precautions, it is in many instances preferable; but whether the twisted or interrupted suture be used, the success of the operation mainly depends, first, on the soft parts being thoroughly freed from the subjacent bone; secondly, on the edges of the fissure being so pared that a good, broad, raw surface is left; and thirdly, on the patient being incessantly watched for three or four days by a skillful nurse, who should support the newly united surfaces by making continuous but gentle pressure on each cheek. I have little confidence in the use of mechanical appliances *after* the operation; for, in nursing the child, they are apt to shift their place, and often do more harm than good. They are, however, invaluable as aids in bringing the two superior maxillæ together if worn *before* operative procedure is undertaken. Again, strapping is of especial service before the operation, but afterwards it cannot be with safety solely relied on."

The context is profusely illustrated, and a very complete index renders the reference to any subject of its contents an easy matter.

F. C. H.

ARTICLE XXI.—SORE THROAT, ITS NATURE, VARIETIES AND TREATMENT; including the Connection between Affections of the Throat and other diseases. By Prosser James, M.D., M.R.C.P., Lecturer on Materia Medica and Therapeutics at the London Hospital; Physician to the Hospital for Diseases of the Throat; late Physician to the North London Consumption Hospital. Third edition. Lindsay & Blakiston, Phila.

This is a small book of about three hundred pages, by the author of the little work on the use of the laryngoscope, which was so favorably spoken of in these pages a few months since. The first edition was published in 1860, and it was at that time, and continued for about three years to be the only English book on the laryngoscope.

The subjects treated of are handled in a concise and lucid manner, which renders the book especially valuable to those physicians who have not the time to make themselves thoroughly familiar with laryngoscopy. The first chapter is devoted to a consideration of the nature and varieties of sore throat; the second to diagnosis of throat affections with the aid of the laryngoscope, and the third to the general therapeutics adapted to this disease. Following them are chapters on inflamed, ulcerated, follicular, aphthous and fungous sore throat. Also chapters on the soft palate, tonsils, pharynx, larynx, laryngeal phthisis, etc., etc. The book contains twelve colored illustrations of diseases of the larynx, and several wood cuts which illustrate the use of the laryngoscope.

The praise we gave the little work on the use of the laryngoscope would apply equally well to this book, but the criticisms on the letter press of the former are fortunately not deserved by this; though we are still left to regret that a book so well written should be illustrated by such poor cuts, however, as we do not expect the exhibition of very high art by the wood engraver, we feel justified in highly recommending the work; for although the cuts are rough, they answer fairly the intended purpose.

E. F. I.

Article XXII.—A Treatise on the Science and Practice of Midwifery. By W. S. Playfair, M.D., F.R .P With notes and illustrations by Robert P. Harris, M.D. Second Amer. from second London Ed. Phil.: H. C. Lea, 1878.

The success of this work, requiring a second edition so soon after the issue of a very large first, is proof sufficient of a popularity that could hardly have been obtained, had it not possessed many points of real excellence. It is not difficult to recognize wherein lies the particular value of the book. Though lacking in explicit details and originality, it is nevertheless a valuable text book for the student of midwifery because of its conciseness. Briefly and clearly written, it presents to the reader a comprehensive history of obstetric science to-day.

The first chapter devoted to "The Bony Pelvis and Pelvic Measurements," is particularly good, and illustrated in a way to compare with any similar work. While in that which relates to conception and generation there is such a prolific exhibition of new terms, that we are afraid the student may lose his way from an imperfect understanding of the matter. One of the best portions of the work is that part of the chapter on forceps, written by the American editor, in which he takes exception to the author's imperative instructions to lay the patient on the left side, and upholds the American method, as well as gives a description of the most popular instruments in this country.

We see here mentioned in terms of praise the forceps of E. Warren Sawyer, of this city. He also writes at considerable length on the Cæsarean operation, dystocia from tetanoid uterine constriction, and intra-venous injection of milk as a substitute for the transfusion of blood. In all of these his style of expression, as well as his plain and logical treatment of the different subjects, compare more than favorably with the corresponding parts of the same chapters written by the author, and make us almost wish that he had written more of the book. The student and the young practitioner will buy this work because it is cheap, compact and comprehensive, even though the older heads will not praise it because they find nothing new in it.

F. C. H—N.

ARTICLE XXIII.—PHYSIOLOGICAL THERAPEUTICS: A NEW THEORY. By Thomas W. Poole, M.D., M.C.P.S. Ontario: Pub. Toronto News Co.

This is a little work of 231 pages. The author has evidently read carefully and with judgment the most noted physiological and therapeutical writers; but whilst noting well the facts they record, and digesting their theories, he has not at all times coincided with them. To illustrate this statement, and at the same time to set forth the author's views in the most concise form, we cannot do better than quote direct from the introduction, four of the six general principles asserted. The burden of proof of these several principles constitute the book.

"1. The muscles and muscular tissues, generally, of the body are endowed with an inherent contractile power of their own, independent of nervous influence; but this contractile power of the muscles is regulated for voluntary purposes through the agency of the nervous system.

"2. The influence exerted by the nervous system in its relation with muscular tissue, is that of a *restraining*, and not that of a compelling power. Nerve force, then, so far from being the ally, is the direct antagonist of muscular contractile power, and the latter displays itself to the best advantage in proportion as the influence of the former is withdrawn.

"3. Electricity is not a stimulus to nerve or muscle. On the contrary, its action is that of a sedative, anæsthetic and paralyser to nerve tissue. It is through this quality of its action that it soothes pain, whilst its tonic effects depend solely on the indirect improvement in nutrition brought about by an infinite number of contractions and relaxations of muscular fiber.

"6. Certain drugs, by modifying the activity of the vaso-motor nerves (increasing their power by nutritive changes in the cells which generate nerve force, or paralysing the nerves themselves, and so arresting their functional activitity) cause an increase or diminution of the caliber of the blood-vessels, and so exert an important influence, not only over the nutrition and temperature of parts, but in controlling congestive and inflammatory processes, and so restoring normal circulatory activity."

The fourth and fifth general principles which we have omitted in the extract, are for the most part embraced in those we have quoted, but apparently put into relief in order to elucidate that part of the subject more fully. We should scarcely be granted space enough to review extensively the line of argument which our author adopts. Suffice it to say that he quotes frequently from Drs. Carpenter, Anstie, Beard and Rockwell, B. W. Richardson, Ringer, Kuss, Klein and numerous others, in order to show the contradictions to which writers are driven in endeavoring to explain the *modus operandi* of electricity and drugs on what may be called the orthodox theory.

The theory which the author suggests, is certainly simple, and it is our opinion that he has succeeded well so far as he has gone in supporting the theory. The style is clear, and whilst it sometimes appears that a repetition frequently occurs, it is really only in appearance. We sincerely recommend the work to all who seek for an intelligent understanding of the modus operandi of the instruments they wield. In prospect of a second edition, we would recommend to the author the "Journal of Physiology," published in England. A thorough study of the experiments so carefully detailed therein, will enable him either to correct himself, or to convince others; perhaps a little of both.

The publishers have scarcely done the author justice.

